# Identifying Antigenic Switching by Clonal Cell Barcoding and Nanopore Sequencing in *Trypanosoma brucei*

**DOI:** 10.21769/BioProtoc.4904

**Published:** 2023-12-20

**Authors:** Abdoulie O. Touray, Tamara Sternlieb, Tony Isebe, Igor Cestari

**Affiliations:** 1 Institute of Parasitology, McGill University, Ste Anne de Bellevue, QC, Canada; 2 Division of Experimental Medicine, McGill University, Montreal, QC, Canada

**Keywords:** Oxford nanopore sequencing, Trypanosomes, Antigenic switching, DNA barcode, VSG-seq, Variant surface glycoproteins, Antigenic variation

## Abstract

Many organisms alternate the expression of genes from large gene sets or gene families to adapt to environmental cues or immune pressure. The single-celled protozoan pathogen *Trypanosoma brucei* spp. periodically changes its homogeneous surface coat of variant surface glycoproteins (VSGs) to evade host antibodies during infection. This pathogen expresses one out of ~2,500 VSG genes at a time from telomeric expression sites (ESs) and periodically changes their expression by transcriptional switching or recombination. Attempts to track VSG switching have previously relied on genetic modifications of ES sequences with drug-selectable markers or genes encoding fluorescent proteins. However, genetic modifications of the ESs can interfere with the binding of proteins that control VSG transcription and/or recombination, thus affecting VSG expression and switching. Other approaches include Illumina sequencing of the VSG repertoire, which shows VSGs expressed in the population rather than cell switching; the Illumina short reads often limit the distinction of the large set of VSG genes. Here, we describe a methodology to study antigenic switching without modifications of the ES sequences. Our protocol enables the detection of VSG switching at nucleotide resolution using multiplexed clonal cell barcoding to track cells and nanopore sequencing to identify cell-specific VSG expression. We also developed a computational pipeline that takes DNA sequences and outputs VSGs expressed by cell clones. This protocol can be adapted to study clonal cell expression of large gene families in prokaryotes or eukaryotes.

Key features

• This protocol enables the analysis of variant surface glycoproteins (VSG) switching in T. brucei without modifying the expression site sequences.

• It uses a streamlined computational pipeline that takes fastq DNA sequences and outputs expressed VSG genes by each parasite clone.

• The protocol leverages the long reads sequencing capacity of the Oxford nanopore sequencing technology, which enables accurate identification of the expressed VSGs.

• The protocol requires approximately eight to nine days to complete.

**Graphical overview**


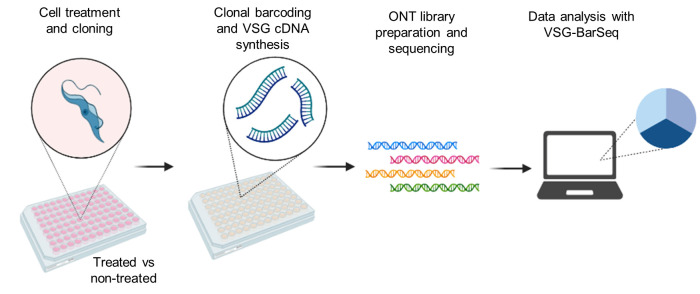


## Background

*Trypanosoma brucei* is a single-celled protozoan parasite that causes African trypanosomiasis and evades the host antibody response by changing its surface coat by antigenic variation (Cestari and Stuart, 2018). *T. brucei* expresses a single variant surface glycoprotein (VSG) gene from one of the 20 telomeric expression sites (ESs) and periodically switches to a different VSG via transcriptional switching between ESs or by VSG gene recombination. *T. brucei* has an extensive repertoire of over 2,500 VSG genes and pseudogenes located in telomeric and sub-telomeric regions of large chromosomes. VSG genes are also found on dozens of mini chromosomes often used for VSG gene recombination. VSG genes are approximately 2 kb in length with conserved C-terminus sequences. VSG recombination can occur by gene or segmental gene conversion, resulting in new mosaic VSG sequences. The mechanisms controlling VSG monogenic expression and switching likely entail multiple processes, including controlling VSG repression and expression via proteins associated with telomeric ESs. Several proteins associate with the telomeric repeats or ESs to regulate VSG gene expression and/or switching, such as the repressor activator protein 1 (Touray et al., 2023), phosphatidylinositol phosphate 5-phosphatase (PIP5Pase) (Cestari et al., 2019), telomeric repeat-binding factor (Jehi et al., 2014), VSG exclusion protein 2 (Faria et al., 2019), and ES body 1 protein (López-Escobar et al., 2022); for a review on additional proteins controlling VSG expression and switching, see (Cestari and Stuart, 2018).

Approaches used to study VSG switching rely on genetic modifications that disrupt the ES DNA sequences by incorporating drug-selectable markers or fluorescent proteins downstream of the promoter sequence and upstream of the VSG gene (Rudenko, 1998; Ulbert et al., 2002; Aitcheson et al., 2005) or by adding exogenous endonuclease sites resulting in DNA breaks (Boothroyd et al., 2009; Glover et al., 2013). However, the ES modifications might disrupt protein binding sites and thus affect VSG switching rates; as an example, RAP1 binds to 70 bp and telomeric repeats flanking ES VSG genes and represses their transcription, and disruption of its binding dramatically increases VSG switching rates (Touray et al., 2023). In addition, the genetic modifications of ESs are laborious and restrict the use of drug-selectable markers available for other genetic changes, such as gene knockout or expression of mutant variants in the cell. Other studies used Illumina RNA-seq to investigate VSG expression at a population level (Mugnier et al., 2015). Although this approach helps to identify expressed VSG genes, many short reads fail to align uniquely to the genome and unambiguously identify and distinguish VSG genes expressed from the extensive repertoire of VSG genes/pseudogenes. Moreover, it does not identify switching cells but expressed VSG genes in the population.

Hence, we sought to develop an approach to track cell-specific antigenic switching without genetically modifying ES sequences in an adaptable high- or medium-throughput fashion. We devised a method to detect VSG switching at nucleotide resolution using clonal cell barcoding and nanopore sequencing. We combine DNA barcoding to identify parasite cell clones and thus track switching and non-switching cells and a broad-spectrum VSG primer for cDNA synthesis to capture all transcribed VSGs in parasite clones. Barcoded samples are multiplexed, and VSG cDNAs are sequenced using Oxford nanopore sequencing, followed by sequencing data analysis using a streamlined VSG-BarSeq pipeline to identify switchers. We performed VSG-BarSeq after a temporary knockdown of PIP5Pase, an enzyme that regulates VSG switching in *T. brucei* (Touray et al., 2023). We found that 99% of the clones switched VSG genes, whereas no switching was detected in the control no-knockdown cell line. The long nanopore reads helped to identify complete VSG sequences and thus track modes of switching. We detected VSG switching by transcriptional and recombination mechanisms, indicating the approach's usefulness in studying antigenic variation. This protocol will enable the efficient study of VSG switching without genetic alterations of the ES. The protocol may be easily adapted to other organisms to study antigenic switching (e.g., *Plasmodium, Giardia*) or clonal expression analysis of large gene families in prokaryotes or eukaryotic cells.

## Materials and reagents

**Biological materials**

1. *Trypanosoma brucei* 427 strain bloodstream forms or conditional null for the gene PIP5Pase derived from the 427 strain (Cestari and Stuart, 2015)

**Reagents**

1. M-MuLV reverse transcriptase (New England Biolabs Ltd, catalog number: M02535)
2. M-MuLV reverse transcriptase 10× buffer (New England Biolabs Ltd, catalog number: B02535)
3. MgCl_2_ (Invitrogen, catalog number: R0971)
4. NucleoMag NGS Clean-up and Size Selection beads (Takara, catalog number: 744970.5)
5. NEBNext end repair module (New England Biolabs Ltd, catalog number: E6050S)
6. Native Barcoding Expansion 1-12 (Oxford Nanopore Technologies, catalog number: EXP-PBC001)
7. 10 M Sodium hydroxide solution (NaOH) (Sigma, catalog number: 1310-73-2)
8. NEBNext Quick Ligation Module (New England Biolabs Ltd, catalog number: E6056S)
9. NEBNext FFPE DNA Repair Mix 24 reactions (New England Biolabs Ltd, catalog number: M6630S)
10. Taq DNA polymerase with ThermoPol buffer (New England Biolabs Ltd, catalog number: M0267S)
11. Deoxyribonucleotide triphosphate (dNTPs) mixture 10 mM (Biobasic, catalog number: DD0056)
12. Agarose (Bioshop Canada Inc., catalog number: AGA002.250)
13. Iscove's modified Dulbecco's medium (IMDM) powder (Life Technologies, catalog number: 12200069)
14. Sodium bicarbonate (Fisher Scientific, catalog number: S233-3)
15. Hypoxanthine (Millipore, catalog number: 4010CBC-25GM)
16. Sodium pyruvate (Wisent Inc., catalog number: 600-110-EL)
17. Bathocuproin sulfonate disodium salt hydrate (Thermo Scientific, catalog number: B22550.MD)
18. L-cysteine hydrochloride monohydrate (Thermo Scientific, catalog number: J14035-22)
19. 2-mercaptoethanol (Sigma, catalog number: M722)
20. Penicillin streptomycin (MP Biomedicals, catalog number: 1670049)
21. Heat-inactivated fetal bovine serum (FBS) (Life Technologies, catalog number:12484-028)
22. Neomycin (G418) (Sigma, catalog number: N6386)
23. Tetracycline hydrochloride (Fisher Scientific, catalog number: BP912-100)
24. Sodium phosphate dibasic anhydrous (Na_2_HPO_4_) (Fisher Scientific, catalog number: S374-500)
25. Sodium phosphate monobasic anhydrous (NaH_2_PO_4_) (Sigma, catalog number: S-0751)
26. Sodium chloride (NaCl) (Fisher Scientific, catalog number: S-271-3)
27. Glucose (Sigma, catalog number: G5400-250G)
28. Sodium citrate (dihydrate) (Fisher Scientific, catalog number: S279-500)
29. Bovine serum albumin (BSA) (Biobasic, catalog number: A500023-0100)
30. Glycerol (Fisher Bioreagents, catalog number: BP229-1)
31. Tris base (Fisher Scientific, catalog number: BP152-5)
32. Glacial acetic acid (Fisher Chemical, catalog number: A38-212)
33. Ethylenediaminetetraacetic acid (EDTA) (Fisher Scientific, catalog number: BP120-500)
34. Trypan Blue solution, 0.4% (Amresco, catalog number: K940-100ML)
35. Ethyl alcohol anhydrous solution (Commercial Alcohols, catalog number: P016EAAN)
36. 96-well plate bacterial total RNA mini-prep super kit (Biobasic, catalog number: BS585-5)
37. Oxford Nanopore Ligation Sequencing Kit (Oxford Nanopore Technologies, catalog number: SQK-LSK109)
38. AMPure XP beads (Beckman Coulter Inc., catalog number: A63880)
39. Phleomycin (Bioshop Canada Inc., catalog number: PEO422.10)

**Solutions**

1. HMI-9 cell culture medium (see Recipes)
2. Drug selectable markers (see Recipes)
  - Neomycin (G-418) stock solution (20 mg/mL)
  - Tetracycline stock solution (5 mg/mL)
  - Phleomycin stock solution (1 mg/mL)
3. Phosphate-buffered saline-glucose (PBS-G) (see Recipes)
4. Bloodstream stabilate freezing solution (see Recipes)
5. 50× Tris acetate EDTA solution (TAE) (see Recipes)
6. 80% Ethanol (see Recipes)

**Recipes**

1. **HMI-9 cell culture medium (1,000 mL)**
  **Table:** 

Reagent	Final concentration	Quantity or Volume
IMDM	n/a	17.7 g
Sodium bicarbonate	0.035 M	3 g
Hypoxanthine	10 mM	10 mL
Sodium pyruvate	100 mM	10 mL
Bathocuproine sulfonate disodium salt hydrate	49.95 µM	28.2 mg
L-cysteine hydrochloride monohydrate	1.04 mM	182 mg
2-mercaptoethanol	14.3 M	14 µL
Nanopure MilliQ water	n/a	879.9 mL
Total volume	n/a	900 mL
  Dissolve the IMDM and salts in 500 mL of water and stir to mix thoroughly. Add 10 mL of hypoxanthine, 10 mL of sodium pyruvate, and 14 µL of 2-mercaptoethanol. Adjust water volume to 900 mL. Filter the medium using a 0.22 µm filter system of 250 mL. Add 100 units of sterile penicillin and 100 µg/mL sterile streptomycin (optional). Complete the media by adding 10% heat-inactivated FBS. Store at 4 °C. The shelf life of the medium is four months.
2. **Drug-selectable markers**
  - Neomycin (G-418) stock solution (20 mg/mL)
    **Table:** 

Reagent	Final concentration	Quantity or Volume
Neomycin (G-418)	n/a	1 g
Nanopure MilliQ water	n/a	50 mL
Total	n/a	50 mL
  - Tetracycline stock solution (5 mg/mL)
    **Table:** 

Reagent	Final concentration	Quantity or Volume
Tetracycline hydrochloride	n/a	0.5 g
Nanopure MilliQ water	n/a	10 mL
Total	n/a	10 mL
  - Phleomycin stock solution (1 mg/mL)
    **Table:** 

Reagent	Final concentration	Quantity or Volume
Phleomycin	n/a	0.05 g
Nanopure MilliQ water	n/a	50 mL
Total	n/a	50 mL
  Dissolve the drugs in water. Filter the dissolved drug solutions using a 0.22 µm syringe filter. Prepare 1 mL aliquots into 1.5 mL Eppendorf tubes in the hood and store the aliquots at -20 °C freezer. Aliquots are stable for at least one year when stored at -20 °C.
3. **Phosphate-buffered saline-glucose (PBS-G) (1,000 mL)**
  **Table:** 

Reagent	Final concentration	Quantity or Volume
Sodium phosphate dibasic anhydrous	0.01 M	1.42 g
Sodium phosphate monobasic anhydrous	0.01 M	1.20 g
Sodium chloride	0.145 M	8.5 g
D-Glucose	0.006 M	1.081 g
Nanopure MilliQ water	n/a	1,000 mL
Total	n/a	1,000 mL
  Dissolve salts in 900 mL of water. Add D-Glucose and stir thoroughly to dissolve it. Adjust the pH to 7.0 and filter sterilize using a 0.22 µm filter. Store at 4 °C. This buffer is stable for three months at 4 °C.
4. **Bloodstream stabilate freezing solution (500 mL)**
  **Table:** 

Reagent	Final concentration	Quantity or Volume
D-Glucose	0.052 M	9.3 g
Sodium chloride	0.036 M	2.1 g
Sodium citrate	0.003 M	0.75 g
BSA	n/a	0.5 g
Glycerol	n/a	75.0 g
Nanopure MilliQ water	n/a	500 mL
Total	n/a	500 mL
  Dissolve the salts in 300 mL of water. Add D-glucose and stir thoroughly to dissolve it. Weigh the volume of glycerol equivalent to 75 g in a separate container and add it to the solution. Adjust the volume of the solution to 500 mL and stir thoroughly. Filter using a 0.22 µm filter. Store it at 4 °C. The solution is stable for six months.
5. **50× Tris Acetate EDTA solution (TAE) (500 mL)**
  **Table:** 

Reagent	Final concentration	Quantity or Volume
Tris base	1 M	121 g
Glacial acetic acid	1 M	28.6 mL
EDTA	0.05 M	50 mL
Nanopure MilliQ water	n/a	421.4 mL
Total volume	n/a	500 mL
  Prepare a stock solution of 0.5 M EDTA in a separate tube by dissolving 18.6 g of EDTA disodium salt in 80 mL of water. Adjust the pH to 8.0 with 10 M NaOH solution. Adjust the volume to 100 mL. Dissolve the Tris base in 300 mL of water and add 28.6 mL of 17.4 M glacial acetic acid and 50 mL of 0.5 M EDTA. Adjust the volume of the solution to 500 mL with water. Autoclave the solution. Store it at room temperature (RT). The solution is stable for at least six months.
6. **80% Ethanol**
  **Table:** 

Reagent	Final concentration	Quantity or Volume
Ethyl alcohol anhydrous solution (100% v/v)	80 %	80 mL
Nanopure MilliQ water	n/a	20 mL
Total volume	n/a	100 mL
  Prepare 80% v/v ethanol by transferring 80 mL of 100% Ethyl alcohol solution to a 100 mL graduated cylinder. Then, add 20 mL of Nanopure MilliQ water. Store at 4 °C. The solution is stable for one month.

**Laboratory supplies**

1. Tissue culture flasks
  - 25 cm^2^ TC-treated T-flask with filter cap (Biobasic, catalog number: SP81136)
  - 75 cm^2^ TC-treated T-flask with filter cap (Biobasic, catalog number: SP81186)
2. Cell culture plates
  - 96-well cell culture plates (WUXI NEST Biotechnology Co., catalog number: 101722BL01)
  - 24-well TC plates, treated (Biobasic, catalog number: SP41135)
3. Sterile pipette tips
  - 1,000 µL pre-sterile barrier tips (Neptune, catalog number: BT100.96)
  - 200 µL pre-sterile barrier tips (Neptune, catalog number: BT200)
  - 10 µL pre-sterile barrier tips (Neptune, catalog number: BT10)
4. 96-well 2 mL deep plate, natural, edge filled, 10 plates/bag (Biobasic, catalog number: BR581-96NS)
5. 96-well deep collection plates (Biobasic, catalog number: 107-E627LA2221)
6. Plate seals (Biobasic, catalog number: BS585-5)
7. 96-well PCR plate (Life Technologies, catalog number: 4346907)
8. Foil sealing film, non-sterile (Celltreat Scientific Products, catalog number: 501535152)
9. Multichannel pipette (Eppendorf, catalog number: 4056991)
10. Heating block (Eppendorf, catalog number: 535028642)
11. Filter system 250 mL 0.2 µm PES (Fisher Scientific, catalog number: FB12566502)
12. 2.0 mL microtubes (UltiDent Scientific, catalog number: 48-C200-CS)
13. 1.5 mL Eppendorf tubes (Eppendorf, catalog number: 0030123611)
14. 100 mL graduated cylinder (Grainger, catalog number: GGS5PTJ5)
15. 100 mL reagent bottle (UltiDent Scientific, catalog number: 170-14170100)

## Equipment

1. Ultrafocused sonicator (Covaris, M220, catalog number: 006168)
2. Magnetic rack for 1.5 mL tubes (Promega, catalog number: PR-Z5342)
3. T100 thermal cycler (Bio-Rad, catalog number: 1861096)
4. Avanti J-E centrifuge (Beckman Coulter, catalog number: JSE02M13)
5. Allegra^TM^ 25R centrifuge, TJ-25 rotor (Beckman Coulter, catalog number: AJC024001)
6. Nanodrop (Nanodrop Spectrophotometer, catalog number: ND-1000)
7. Microcentrifuge (Eppendorf, catalog number: EP5401000137)
8. Water system ultrapure (Nanopure MilliQ water) (Millipore Synergy, catalog number: F1CA45528 A)
9. Electrophoresis unit: Thermo Scientific Power Supply 400 mA 300 V (Fisher, catalog number: S65533Q)
10. Biological safety cabinet (NuAire Biological Safety Cabinet Class II Type AIB3, catalog number: NU-425-300)
11. CellDrop^TM^ automated cell counter (DeNovix Cell Drop FL Fluorescence Cell Counter)
12. HEPA CO_2_ incubator (Thermo Electron Corporation; Forma Series II, Water Jacketed, catalog number: 308606-30529)
13. Microscope (Nikon, model: Eclipse TS100, catalog number: 302115)
-80 °C freezer (New Brunswick Ultra-Low Temperature Freezer U101 Inova, catalog number: U9420-0000)
15. MinION Mk1C (Oxford Nanopore Technologies, catalog number: MIN-101C)
16. Flongle Flow Cell (R9.4.1) (Oxford Nanopore Technologies, catalog number: FLO-FLG001)
17. Rotator (ManSci Inc., catalog number: A706514)
18. Milli-Q IQ 7000 purification system (Millipore Sigma, catalog number: ZIQ7000T0C)

## Software and datasets

1. Minimap2, version 2.24 (Li, 2018) (https://github.com/lh3/minimap2)
2. Samtools, version 1.17 (Danecek et al., 2021) (https://github.com/samtools/samtools)
3. DeepTools, version 2.0 (Ramírez et al., 2016) (https://deeptools.readthedocs.io/en/develop/index.html)
4. Subread, version 2.0.3 (Liao et al., 2014) (http://subread.sourceforge.net/featureCounts.html)
5. Rcgrep, version 0.1 (https://github.com/dib-lab/rcgrep)
6. Integrative Genomics Viewer (IGV), version 2.16.2 (Robinson et al., 2011) (https://software.broadinstitute.org/software/igv/)
7. VSG-BarSeq (this work), version 1.0.1 (https://github.com/cestari-lab/VSG-Bar-seq)

## Procedure

- **Parasite treatment and cloning**
  We recommend determining cell treatment conditions before starting this protocol. The conditions used in this protocol were optimized for *T. brucei* bloodstream forms of the 427 strain or conditional null (CN) cells derived from the single-marker 427 strain (Cestari and Stuart, 2015). The treatment described here is the knockdown for 24 h of the *T. brucei* gene encoding PIP5Pase, which results in high rates of VSG switching (Touray et al., 2023). The PIP5Pase CN cell line is grown in G418 and phleomycin to maintain the selection of the tetracycline-inducible system. Tetracycline is added to induce expression of the PIP5Pase gene under the control of a procyclin promoter and a tetracycline operator.
  1. Grow 5 mL of *T. brucei* cells seeded at 1.0 × 10^4^ cells/mL in HMI-9 medium supplemented with 2 µg/mL G418, 2.5 µg/mL phleomycin, and 500 ng/mL tetracycline in a 37 °C incubator with 5% CO_2_ for 24 h or until it reaches mid-log growth (~1.0 × 10^6 ^cells/mL). Throughout this protocol, cell growth will be as described above unless otherwise stated. Cells’ doubling time should be approximately 5.5–6 h and viability approximately 90%–95%. Avoid overgrowing cell culture (>1.5 × 10^6 ^cells/mL) because it will affect cell viability.
  2. Transfer the 5 mL cell culture to a 15 mL Falcon tube and centrifuge at 3,500× *g* for 5 min at RT. Discard the supernatant.
  3. Resuspend the pellet in 10 mL of PBS-G pre-warmed at 37 °C and then centrifuge the cells as in step A2. Discard the supernatant.
  4. Repeat step A3 three times to ensure complete removal of tetracycline.
  5. Split the cells into two 5 mL cell culture flasks (treated and non-treated groups), seeding each culture at 1.0 × 10^4^ cells/mL in HMI-9 medium with 2 µg/mL G418 and 2.5 µg/mL phleomycin.
  6. Add 500 ng/mL tetracycline to the non-treated flask (Tet +, control) and no tetracycline to the treatment flask (Tet -, knockdown) and grow the cells for 24 h.
  7. Add 500 ng/mL tetracycline to the treatment flask (Tet -, knockdown). No additional tetracycline is required for the non-treated flask (Tet +, control). Quantify the cell concentration and viability of both the treatment and control groups by mixing 10 µL of cell culture and 10 µL of 0.4% Trypan blue staining. Add 10 µL to the CellDrop^TM^ cell counter to obtain viability and cell concentration. Cell concentration should be approximately 1.0 × 10^5^ cells/mL, and viability should be >90%.
  8. Add 9 mL of HMI-9 medium supplemented with 500 ng/mL tetracycline to a 50 mL Falcon tube. Repeat the procedure to have three flasks for the treatment group and three for the non-treatment group.
  9. From a starting cell concentration of 1.0 × 10^5^ cells/mL (from A7 above), gently mix the cells by flicking the flasks five times and transfer 1 mL of the culture to the Falcon tube 1 containing 9 mL of HMI-9 media (1 in 10 dilutions) to obtain a cell concentration of 1 × 10^4^ cells/mL.
  10. From the Falcon tube 1 (1 × 10^4^ cells/mL) culture, transfer 1 mL to Falcon tube 2 to obtain a cell concentration of 1 × 10^3^ cells/mL (1 in 10 dilutions). Repeat the same for Falcon tube 3 to obtain a cell concentration of 1 × 10^2^ cells/mL. Perform the procedure for control and treated groups.
  11. Transfer 7 mL of the culture from Falcon tube 3 to a 75 cm^2^ cell culture flask containing 63 mL of HMI-9 medium supplemented with 2 µg/mL G418, 2.5 µg/mL phleomycin, and 500 ng/mL tetracycline, to obtain a cell concentration of 10 cells/mL (1 in 10 dilutions).
  12. Transfer 60 mL of the diluted cell culture from step A11 to a 75 cm^2^ cell culture flask and add 140 mL of HMI-9 medium supplemented with 2 µg/mL G418, 2.5 µg/mL phleomycin, and 500 ng/mL tetracycline (3 in 10 dilutions) to obtain a final cell concentration of 3 cells/mL.
  13. Aliquot the diluted parasite culture (3 cells/mL) onto ten 96-well cell culture plates (10 plates per treatment group) using a multichannel pipette. Transfer 200 µL to each well so that the probability of obtaining one single cell per well (200 µL) is approximately 30%, i.e., one cell per well for a third of the wells of a 96-well cell culture plate. Ensure the cells are well mixed by gently swirling the parasite cultures while pipetting.
  14. Grow the cells in 96-well cell culture plates in an incubator for 5–7 days.
  15. Check the 96-well cell culture plates under a microscope to identify parasite clones. Approximately 30% of the wells should contain parasites (see Note 1). We recommend checking each well for parasite clones starting from day 5 up until day 7 post seeding.
  16. Transfer 200 µL of parasite clones onto 24-well TC plates and add 1.8 mL of fresh HMI-9 with 2 µg/mL G418, 2.5 µg/mL phleomycin, and 500 ng/mL tetracycline.
  17. Grow the clones in the 24-well TC plates for 24 h to increase the number of cells for RNA extraction.
  18. (Optional) Aliquot 600 µL of each clonal population onto new 96-well deep collection plates and add 600 µL of freezing solution. Freeze the parasites at -80 °C for short-term storage (2–3 weeks) or liquid nitrogen for long-term storage.
  19. The remaining 1.4 mL of the clonal parasite cultures are used for RNA extraction.
- **RNA extraction**
  1. Transfer 1.4 mL of each clonal culture (approximately 1.4 × 10^6 ^cells) into 96-well deep collection plates, centrifuge at 3,500× *g* for 5 min at RT, and pour off the supernatant (see Note 2).
  2. Add 350 µL of buffer Rlysis-BG (provided in the 96-well plate bacterial total RNA mini-prep super kit) to each well and resuspend the cells by pipetting up and down five times.
  3. Thoroughly seal the plate with a sealing film to prevent cross-contamination of the samples and immediately mix the samples by inverting the plate three times.
  4. Briefly spin the plate at 1,000× *g* for 30 s to collect the solution to the bottom of the wells.
  5. Add 175 µL of absolute ethanol to each well, tightly seal the plate with a new sealing film, and mix thoroughly by inverting five times.
  6. Place the EZ-10 96-well plate (filtration column plate provided with the 96-well plate bacterial total RNA mini-prep super kit) on top of a new 96-well deep collection plate and transfer the lysate from step B5 into the columns on the EZ-10 96-well plate.
  7. Centrifuge the plate at 5,000× *g* for 2 min at RT and discard the flowthrough.
  8. Place the EZ-10 96-well plate back on the deep cell collection plate and add 500 µL of universal GT solution (provided in the kit) to each column.
  9. Centrifuge the plate at 5,000× *g* for 1 min at RT and discard the flowthrough.
  10. Place the EZ-10 96-well plate back on the deep well collection plate and add 500 µL of universal NT solution (provided in the kit) to each column.
  11. Centrifuge the plate at 5,000× *g* for 1 min at RT and discard the flowthrough.
  12. Place the EZ-10 96-well plate back on the deep well collection plate and centrifuge the column at 5,000× *g* for 2 min at RT to ensure complete removal of the residual ethanol.
  13. Place the EZ-10 96-well plate into a new deep-well storage plate (provided in the kit), add 30 µL of RNase-free water (supplied in the kit), and then incubate the plate at RT for 5 min.
  14. Centrifuge the plate at 5,000× *g* for 1 min at RT to elute the RNA solution.
  15. Quantify the recovered RNA by measuring 1 µL of the RNA solution at 260 nm from approximately 10 random wells using a NanoDrop. This will help estimate the isolated RNA concentrations.
  16. Tightly seal the plate with an adhesive cover and keep it on ice (or at -80 °C) until the RNA samples are ready for cDNA synthesis.
- **VSG-enriched cDNA synthesis and barcoding**
  This step requires a combination of primers (Figure 1) to barcode each clonal cell population cDNAs with a unique eight-nucleotide sequence for their identification during sequencing analysis. It will also provide an adapter sequence for DNA sequencing library preparation. The forward Ad-SL (5′-TTTCTGTTGGTGCTGATATTGCacagtttctgtactatattg-3′) primer is universal and includes an Oxford nanopore adaptor sequence (capital letters) followed by a sequence (small letters) that hybridizes to mRNA splice leader sequence, a 39-nt sequence added to the 5′ of all trypanosomes’ mRNAs. The reverse Ad-3endVSG primer (5′-TACTTGCCTGTCGCTCTATCTTCXXXXXXXXgtgttaaaatatatc-3′) contains an Oxford nanopore adaptor sequence (capital letters) followed by eight-nucleotide long variable sequence (barcode) unique to each clone and a sequence pairing with the conserved 3′-end of VSG mRNAs, which encodes the C-terminus of VSG proteins (Mugnier et al., 2015). See Supplementary information for the complete primer list. We recommend preparing a working primer solution containing a mix of both primers at 10 µM in a 96-well plate.
  1. Take 4 μL of primer mix from the working primer solution plate and transfer it to each well of a new 96-well PCR plate using a multichannel pipette.
  2. Add 1 μL of 10 mM dNTPs mix onto each well of the same 96-well PCR plate.
  3. Thaw the RNA samples (from step B16) on ice if frozen at -80 °C and add 5 μL of the samples, keeping the same orientation in the 96-well PCR plate as the original RNA plate. Mix the solutions by gently pipetting up and down five times and spin down the plate briefly at 1,000× *g* for 30 s at 4 °C.
  4. Incubate the plate at 65 °C for 5 min in a thermocycler and then transfer the plate immediately to ice.
  5. Prepare cDNA synthesis master mix by adding 2 μL of MuLV 10× Buffer, 5 U of M-MuLV Reverse Transcriptase, and 8 U of RNase inhibitor (supplied in the MuLV reverse transcriptase kit) and adjust the reaction volume to 10 μL per reaction using nuclease-free water.
  6. Aliquot 10 μL of the cDNA synthesis master mix onto each well of the 96-well plate containing the RNA, dNTPs, and primer mix.
  7. Thoroughly seal the plate using an adhesive plate sealing film, spin down at 1,000× *g* for 30 s, and incubate at 42 °C for 2 h and then 65 °C for 20 min in a thermocycler.
  8. Store the synthesized cDNA samples at -80 °C or proceed to library preparation.
- **Oxford nanopore library preparation and DNA sequencing**
  1. Combine the synthesized cDNA samples from each well (from step C8) into one 1.5 mL microcentrifuge tube and mix gently five times. Avoid forming bubbles.
  2. Prepare 10 barcoding PCR reactions containing 5 μL of 10× ThermoPol buffer, 400 μM dNTPs mix, 500 nM barcode primer mix (Native Barcoding Expansion 1-12), 2 U of Taq DNA Polymerase, and 750 nM MgCl_2_. Add 3 μL of the pooled cDNA and adjust the final reaction volume to 50 μL with nuclease-free water.
  3. Perform the PCR reaction in a thermocycler at 95 °C for 10 min, then 22 cycles at 95 °C for 1 min, 62 °C for 1 min, and 68 °C for 3:30 min, and a final extension at 68 °C for 10 min (see Notes 3 and 4).
  4. Pool together all PCR amplicons in one 1.5 mL Eppendorf tube to obtain a final volume of 500 μL. Add 350 μL (0.65× beads to sample ratio) of NucleoMag NGS Clean-up and Size Selection beads to clean up the DNA.
  5. Incubate beads/samples in a rotator (60 rpm) for 10 min at RT.
  6. Spin down the tube briefly at 1,000× *g* for 30 s at RT, place it on a magnetic rack, and incubate for 1 min at RT. Pipette off and discard the supernatant without disturbing the beads.
  7. Add 350 µL of freshly prepared 80% ethanol to the beads gently. Avoid disturbing the beads or taking the tube out of the magnetic rack. The volume of the 80% ethanol to add per wash should be equal to the sum of the total PCR amplicon volume and added NucleoMag NGS beads volume (e.g., 500 µL of PCR reaction + 350 µL of NucleoMag NGS magnetic beads = 850 µL of the mix, then 850 µL of 80% ethanol).
  8. Incubate the mix for 1 min on the magnetic rack at RT. Collect and discard the 80% ethanol. Repeat steps D7 and D8.
  9. Centrifuge the tube at 1,000× *g* for 30 s at RT. Place the tube back on the magnetic rack and remove any residual 80% ethanol from the tube.
  10. Remove the tube from the magnetic rack and let the beads air dry for 10 min at RT.
  11. Resuspend beads in 61 μL of nuclease-free water, mix gently by flicking the tube or gently pipetting up and down five times, and incubate for 5 min at RT.
  12. Place the tube back on the magnetic rack and incubate for 1 min at RT.
  13. Collect and transfer the clear supernatant containing the eluted DNA into a new labeled tube. Discard the tube containing the beads.
  14. Measure and assess the concentration and purity of the eluted DNA by quantifying 1 µL of the DNA sample using NanoDrop. The DNA yield ranges from approximately 30 to 60 ng/μL with 260/280 and 260/230 absorbance ratios of approximately 1.8 and 2.0, respectively.
  15. Prepare 1 μg of DNA in 47 μL of nuclease-free water in a 0.2 mL thin-walled PCR tube and then add the following: 3.5 μL of NEBNext FFPE DNA repair buffer and 2 μL of NEBNext FFPE DNA repair mix (from NEBNext FFPE DNA Repair Mix), 3.5 μL Ultra II End-prep reaction buffer and 3 µL Ultra II End-prep enzyme mix (from NEBNext end repair module), and 1 μL of DNA CS (provided in the Oxford Nanopore Ligation Sequencing kit).
  16. Mix the solutions by pipetting up and down gently five times and incubate for 60 min at 20 °C in a thermocycler.
  17. Add 39 μL of NucleoMag NGS Clean-up and Size Selection beads to the end-repaired DNAs (0.65× beads to samples ratio) to clean up and follow steps D5–D13. Elute the DNA in 61 μL of nuclease-free water.
  18. Quantify 1 µL of the eluted DNA using NanoDrop. Approximately 90% of the DNA is expected to be recovered.
  19. Transfer the remaining 60 µL of end-repaired DNA volume from step D18 (0.5–1 μg) to a 0.2 mL thin-walled PCR tube and then add the following components: 25 μL of ligation buffer (LNB), 10 μL of NEBNext Quick T4 DNA ligase (from NEBNext Quick Ligation Module), and 5 μL of Adapter mix F (AMX-F) (LNB and AMX-F are components of the Oxford Nanopore Ligation sequencing kit). Mix by gently pipetting the reaction up and down five times, then briefly spin it down.
  20. Incubate the reaction for 2 h at 20 °C in a thermocycler.
  21. Transfer the ligation reaction mix from step D20 to a new 1.5 mL Eppendorf tube and add 40 μL of well resuspended AMPure XP beads to the reaction (0.4× beads to sample ratio). Mix by gently pipetting up and down five times.
  22. Incubate the mix on a rotator (60 rpm) for 10 min at RT.
  23. Briefly centrifuge the sample at 1,000× *g* for 30 s and place the tube on a magnetic stand for 1 min at RT or until the supernatant is clear and colorless.
  24. Remove and discard the supernatant without disturbing the beads.
  25. Resuspend the beads in 250 μL of Short Fragment Buffer (SFB) (provided in the Oxford Nanopore Ligation Sequencing kit) and mix gently by flicking the tube five times to wash the beads.
  26. Briefly centrifuge the tube at 1,000× *g* for 30 s. Transfer the tube to a magnetic stand to pellet the beads. Remove and discard the supernatant without disturbing the beads.
  27. Repeat steps D25 and D26.
  28. Briefly centrifuge the tube at 1,000× *g* for 30 s. Place it on the magnetic stand and remove any residual supernatant. Air dry the beads at RT and ensure that they do not dry to the point of cracking. Ten minutes is sufficient for the beads to dry. Extended drying of the beads for 20–30 min might cause them to crack.
  29. Remove the tube from the magnetic stand and resuspend the beads in 12 μL of Oxford nanopore Elution Buffer (EB) (provided in the Oxford Nanopore Ligation Sequencing Kit) by gently pipetting up and down.
  30. Briefly centrifuge the tube at 1,000× *g* for 30 s and incubate for 10 min at RT.
  31. Place the tube back onto the magnetic stand for 1 min and then remove and transfer the eluate containing the DNA library into a new 1.5 mL Eppendorf tube. Dispose of the beads.
  32. Quantify 1 µL of the eluted library DNA sample using a NanoDrop. Expect a DNA library yield of approximately 10–40 ng/μL (i.e., 200–1,500 fmol) of DNA.
  33. Load 3–20 fmol of the prepared library into the Flongle Flow Cell (R9.4.1) and sequence the DNA according to the manufacturer's instructions.
    **Figure 1.**
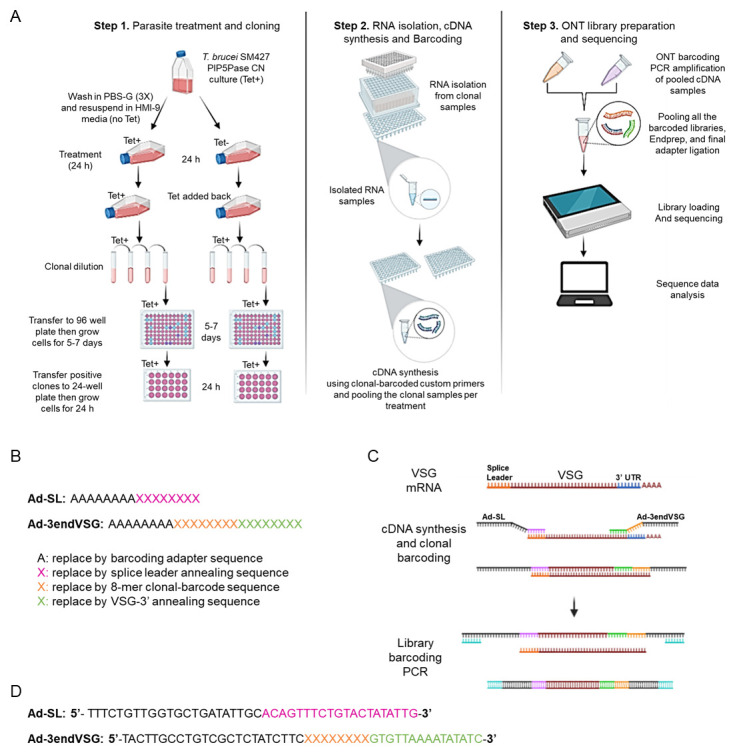
Schematic representation of clonal cell barcode and nanopore sequencing protocol. (A) Diagram of the clonal cell barcoding, nanopore sequencing workflow showing the parasite treatment, and cloning (Step 1), RNA isolation from the individual clones, cDNA synthesis, and clonal barcoding (Step 2), and Oxford nanopore technology (ONT) library preparation, sequencing, and data analysis using VSG-BarSeq script (Step 3). (B) Scheme of the forward Adaptor Splice Leader (Ad-SL) primer and the reverse Adaptor-barcode-3′-end VSG primer (Ad-3endVSG) used for cDNA synthesis and clonal cell barcoding. (C) Diagram describing the cDNA synthesis, clonal barcoding, ONT library barcoding, and PCR amplification. (D) Sequences of the Ad-SL and Ad-3endVSG annealing primers used for cDNA synthesis and clonal cell barcoding (see the annealing regions in C). The sequences in black correspond to ~20 bp nanopore barcode adapter sequences, and the sequences in pink and green correspond to the forward Ad-SL and reverse Ad3endVSG primers, respectively. The Ad-SL pairs to the mRNA splice leader sequence, while the reverse Ad3endVSG primer pairs to the conserved 3′-end of VSG mRNAs. The 8-nucleotide long variable sequence (clonal barcode) unique to each clone is shown in orange and depicted here as X. A complete list of primers is available in Supplementary Information. PBS-G: phosphate-buffered saline (PBS)-glucose; ONT: Oxford Nanopore Technology; PIP5Pase CN: phosphatidylinositol 5-phosphatase (PIP5Pase) conditional null.

## Data analysis

- **Computational analysis of the sequenced library**
  The data analysis described here was performed using a Linux operating system (Ubuntu). The computational resources required will vary depending on the data available for analysis. The Oxford nanopore sequencer will generate fast5 files, which are basecalled to fastq files using the Guppy tool integrated in the MinKNOW software (https://nanoporetech.com/). The fastq files are the input dataset in the analysis shown here. We developed a computational pipeline for the sequencing analysis and detection of VSG switching (Figure 2). The pipeline is run via the vsg-barseq.sh script. A fastq file with 100,906 reads was analyzed using 10 threads and 4 GB of memory and completed in 5 min. The pipeline takes the DNA sequences generated by the nanopore sequencing in fastq format and splits them into subfiles according to the cell barcodes (eight mers) used for DNA-seq library preparation. The output fastq files are named by their barcodes, e.g., CCATGCAT.fastq. A split summary file (.txt) is generated and shows the number of reads per barcode file, the total count of reads analyzed, and the total amount of reads containing barcodes (see Note 5). Sequences from each file are then mapped to the organism genome (here, *T. brucei* 427 strain) using minimap2, outputting .sam files. The alignments are then filtered using Samtools to remove supplementary alignments and to keep alignments with a mapping quality score (mapQ) ≥ 20. The resultant .sam files are sorted and indexed with Samtools resulting in sorted.bam and bam.bai files, respectively.
  The alignments are counted with featureCounts (package Subread). The top mapped reads, which correspond to the expressed VSG, are selected and compiled in a single tab-delimited file (topmapped.txt) while keeping the original output from featureCounts, which serves for analysis of other genes identified during alignment. The analysis of other identified genes is part of the quality control process. It typically shows genes with low counts reflecting low background noise from library preparation resulting primarily from sequences derived from splice-leader cDNA synthesis (Figure 3E).
  The vsg-barseq.sh is open code and available on GitHub (https://github.com/cestari-lab/VSG-Bar-seq). The script takes six arguments:
  1) Directory of all files (output folders will be created in this directory)
  2) Directory of fastq files
  3) Directory of barcode.txt files (barcode.txt file has one barcode sequence per row)
  4) Directory of genome file in fasta format
  5) Directory of gene transfer format file, i.e., gtf format
  6) Number of threads to be passed to minimap2, samtools, and featureCounts (we recommend eight or more)
  Example of barcode.txt file. Keep one barcode sequence per row.
  CCGTTAGG
  CCAACTAG
  CAGGGCAG
  CGCAGAAG
  If the barcoded.txt file is generated using Windows operational system, we recommend converting from dos to Unix format before executing the analysis. The tools minimap2, samtools, subread, and rcgrep are required to run the script. After installing and/or loading the required tools, run the script as indicated below:
  sh vsg-barseq.sh path/to/directory path/to/fastq path/to/barcodes path/to/genome path/to/gtf nthreads
  See the example below using the structure folder exemplified (directories in bold).
  sh vsg-barseq.sh \
  c/user/vsg-barseq \
  myfastq \
  barcodes \
  genomefiles/genome.fasta \
  genomefiles/genome.gtf \
  8
  Note that *T. brucei* genome (.fasta) and features (.gff, general feature format) can be downloaded from TritrypDB (https://tritrypdb.org/). TriTrypDB does not provide .gtf files, but .gff files can be used to generate .gtf. We recommend using the gffread tool (https://github.com/gpertea/gffread), also available via Galaxy tools (https://usegalaxy.org/). The command below indicates how to generate a gtf file from a gff file after installing gffread tools.
  gffread annotation_file.gff -T -o annotation_file.gtf
  *T. brucei* barcodes and test fastq data files are also available for download at https://github.com/cestari-lab/VSG-Bar-seq to test the script.
  Directory structure of input files required to run the script:
**c/user/vsg-barseq/**
  ----**myfastq**/mydata.fastq
  ----**barcodes**/barcode.txt
  ----**genomefiles**/genome.fasta, genome.gtf
  After running the script, the following output directories and files are created:
**c/user/vsg-barseq/**
  ----**result_split_fastq/**CCGTTAGG.fastq, CCAACTAG.fastq, CAGGGCAG.fastq, CGCAGAAG.fastq, split_summary.txt
  ----**result_mapcount/**
  ----**sam/**CCGTTAGG.sam, CCAACTAG.sam, CAGGGCAG.sam, CGCAGAAG.sam,
  ----**bam/**CCGTTAGG.bam, CCAACTAG.bam, CAGGGCAG.bam, CGCAGAAG.bam
  ----**sorted_bam/**CCGTTAGG_sorted.bam, CCGTTAGG_sorted.bam.bai, etc.
  ----**counts/**CCGTTAGG.txt, CCGTTAGG.txt.summary, etc.
  ----**topmap/**topmapped.txt
  The result file topmapped.txt will have information on gene id (first row, in bold below), chromosome (or contig), nucleotide position (start, end), strand (+/-), gene length in nucleotides, number of counted alignments for the gene (seventh row, in bold below), and path to the original file containing all alignment counts for the corresponding clone; the eight mers barcode identifies the cell expressing the VSG. Each row represents a cell clone, and the gene id indicates the VSG expressed by the cells in the clonal population.
  **Tb427_000016000** BES1_Tb427v10 75570 77000 + 1431 **461** /path/to/counts/CCGTTAGG.txt
  **Tb427_000016000** BES1_Tb427v10 75570 77000 + 1431 **363** /path/to/counts/CCAACTAG.txt
  **Tb427_000008000** BES12_Tb427v10 45278 46651 + 1374 **310** /path/to/counts/CAGGGCAG.txt
  **Tb427_000284800** Chr2_5A_Tb427v10 197226 198701 + 1476 **840** /path/to/counts/CGCAGAAG.txt
  **Figure 2.**
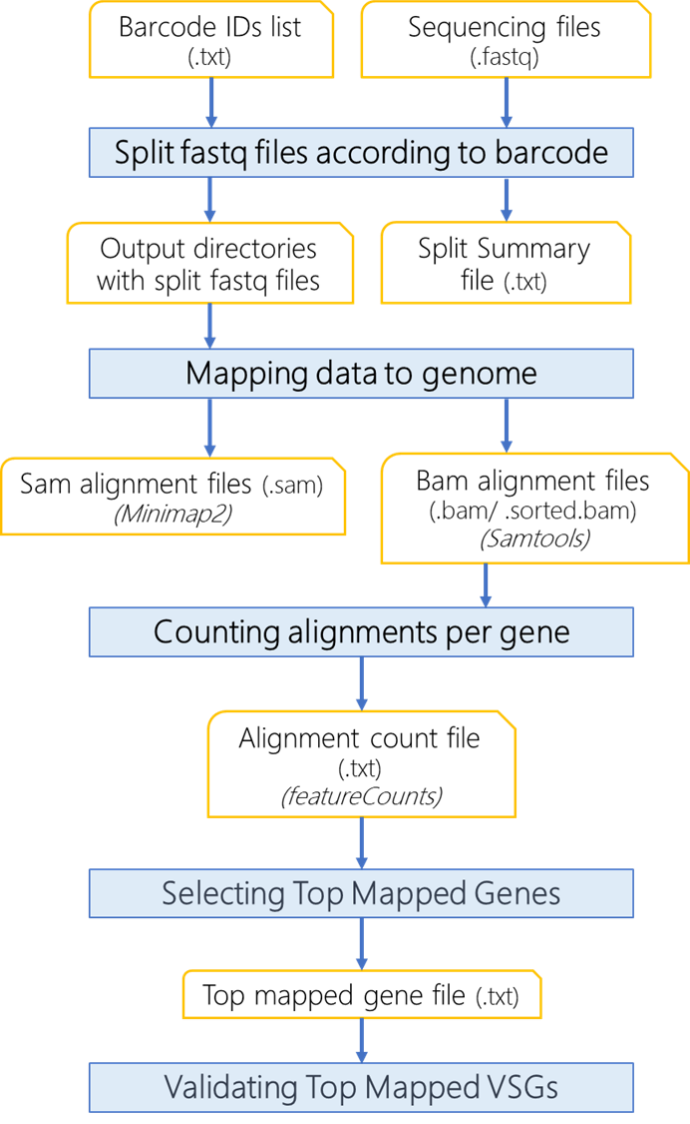
Flowchart of computational analysis using VSG-BarSeq. Multiplexed reads from clonal VSG-seq are split into clone-specific reads based on eight mers barcode. Reads are aligned to the genome using minimap2 and filtered with Samtools to remove supplementary and secondary alignments and keep alignments with mapQ ≥ 10. Then, it counts the alignments per gene and reports the top alignment per file, corresponding to the expressed variant surface glycoproteins (VSG) gene.
- **Anticipated results**
  The analysis of parasite VSG switching starts with treating the cultures to induce the switching of the VSG gene. The treatment conditions and outcomes might differ for different cell lines, so they may need to be optimized. Here, the treatment was the temporal knockdown (24 h) of the PIP5Pase gene, which results in high rates of VSG switching (Touray et al., 2023). VSG switching occurs by alternating transcription between ESs or recombining VSG genes within ESs (Figure 3A). The parasite culture was diluted to obtain approximately 30% of clones from a 96-well plate. We recommend optimizing the dilution of the cells after the treatment and checking cell viability before the VSG-BarSeq experiment. After cDNA synthesis and library amplification with ONT-barcoding primers, we recommend analysis of the product in agarose gel. We often obtain a PCR fragment smear ranging from 200 to 2,000 bp (Figure 3B).
  **Figure 3.**
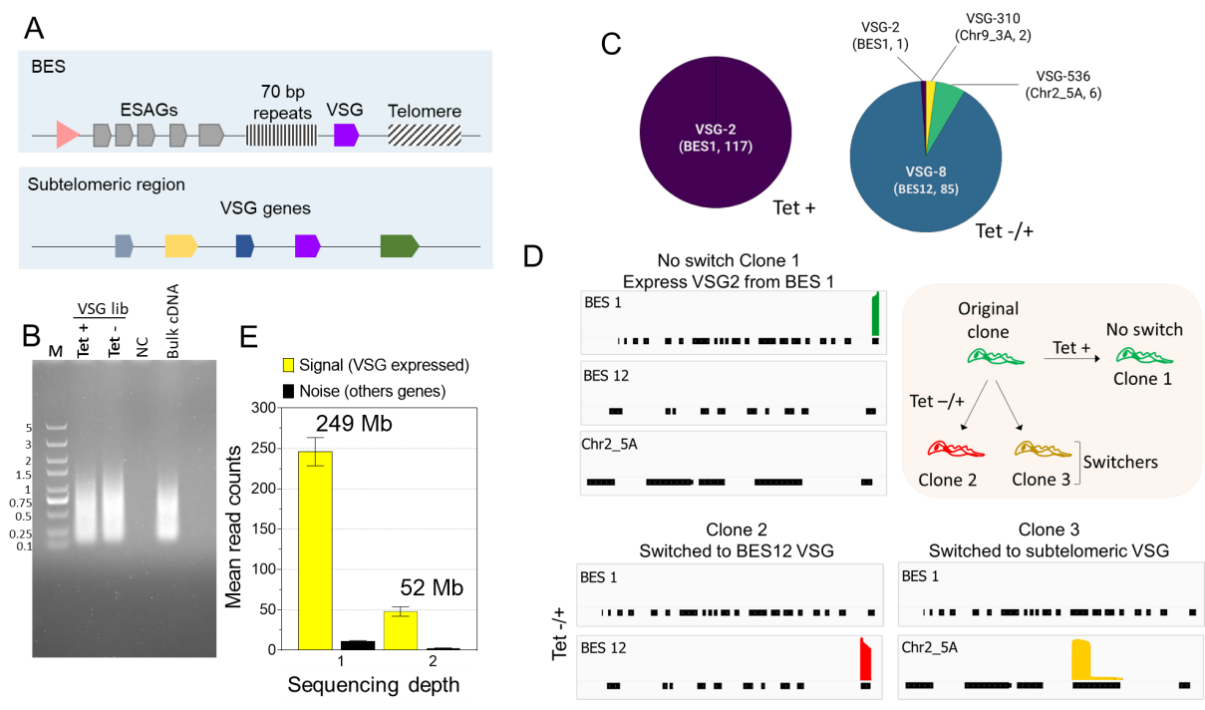
Analysis of variant surface glycoproteins (VSG) switching after PIP5Pase knockdown using VSG-BarSeq. (A) Diagram of bloodstream-form expression sites (ESs) (BES) and sub-telomeric regions containing VSG genes used for recombination. The pink arrowhead represents a BES promoter. VSG genes are transcribed from BESs only. (B) Thirty-five cycles of quality-control PCR amplification of pooled clonal VSG barcoded cDNAs. PCR was amplified with ONT barcode primers. NC: negative control, M: 5 kb DNA ladder. (C) VSG genes expressed by clones of *T. brucei* without PIP5Pase knockdown (Tet +) and after temporary knockdown for 24 h followed by PIP5Pase re-expression and cloning for 5–7 (Tet -/+). Annotated names and corresponding chromosomes or BES identify VSG genes. The number of clones analyzed is indicated in parentheses. (D) Read coverage plots of example clones from Tet + or Tet -/+ treatment groups. The diagram on the top right summarizes the experiment and results—all clones derived from an original clone expressing VSG2 (BES 1). Graphs show read coverage of expressed VSGs on the same scale. (E) Signal-to-noise ratios of two different VSG-BarSeq libraries differing in sequencing depth. Sequencing depth is shown above plot bars as the total reads by the mean read length. Mb: megabases.
  After library preparation, we usually obtain a library concentration ranging between 10 and 40 ng/μL with 260/280 and 260/230 ratios of ~1.8 and 2.0, respectively. Library concentrations below 5 ng/µL with considerable deviations from the above 260/280 and 260/230 ratios usually result in inferior quality and low throughput sequencing. Analysis of the sequencing using the vsg-barseq.sh script typically results in 70%–99% genome mapping. Figure 3C and 3D show the results of VSG switching in *T. brucei* after the temporary knockdown of PIP5Pase. Analysis of 117 clones from the control (no knockdown) showed that none of the cells switched VSGs (Figure 3C). However, after 24 h temporary knockdown, there were 93 switchers out of 94 clones analyzed. There was a preference for the cells to switch to VSG8 in the BES12, suggesting transcriptional switching. Moreover, switching to VSGs from sub-telomeric regions (Chr2_5A and Chr9_3A) was also detected, indicating switching by recombination (Figure 3C and D). The analysis does not require a significant amount of RNAs or sequencing throughput since the experimental setup relies on selecting VSG mRNAs, which are highly abundant (Cestari and Stuart, 2015), and on the analysis of clones rather than heterogeneous cell populations. Analysis of the expressed VSG (signal) compared to other genes (noise) showed a high signal-to-noise ratio, and increasing sequencing depth improved the signal without significantly increasing noise levels (Figure 3E). Although DNA sequencing can be costly, the amount of total RNA for cDNA synthesis per clone in this protocol is minimal (5–20 ng), and sequencing depth required for analysis can be obtained from Oxford nanopore flongle flow cells, which typically produces 500–1,000 megabases of DNA sequencing (i.e., ~100,000 reads per group), thus reducing experimental costs. The results show the method's utility in identifying VSG switching events using multiplexed clonal cell barcodes. We anticipate that, with minimal modifications of the primers used for the target sequences, the approach can be applied to other gene families or other cell types, including var genes in *Plasmodium sp.*, variant surface proteins in *Giardia* sp., as well as mucins and mucin-associated proteins in *Trypanosoma cruzi*. Analogously, the approach can be extended to other organisms or cell lines, including mammalian cells, e.g., T-cell or B-cell repertoire analysis.

## Validation of protocol

This protocol has been used and validated in the following article:

- Touray et al. (2023). A PI(3,4,5)P3-dependent allosteric switch controls antigenic variation in trypanosomes. eLife 12: RP89331 (Figure 1F, and Figure 1—figure supplement 1, panel A, B, C, D).

## General notes and troubleshooting

**General notes**

1. If more than 40% of the wells in a 96-well plate are positive, the parasites might not be clonal. We recommend optimizing the dilution of the parasites to obtain approximately a third of the wells from a 96-well plate containing growing parasites.
2. We recommend performing a quality control PCR prior to cDNA amplification. Use the same conditions indicated in step D3 but for 35 cycles. Then, analyze 30 µL the amplicons on 1% agarose/TAE electrophoresis gel with 5 µL of Ecostain at 85 Volts for 45 min and visualize using a gel imager. A smear migrating at 200–2,000 bp should be expected in the agarose gel (Figure 3B).
3. We recommend the volume of pooled cDNA sample added to the PCR reaction to be less than one-tenth of the total PCR reaction volume.
4. Reverse transcriptase enzymes are known to inhibit PCR, particularly at low template concentrations (Chandler et al., 1998). Therefore, adding more cDNA to the PCR reactions usually results in non or very little amplification.
5. The split summary file helps to identify the splitting of reads into multiple barcode fastq files. If the number of reads with barcodes is larger than the total number of reads, it is indicative that some reads are included in more than one barcode fastq file. Primers with longer barcodes than eight mers could be used.

## Supplementary information

The following supporting information can be downloaded here:

1. Table S1. List of primer sequences indicated in the VSG-BarSeq protocol.
